# Indian Journal

**Published:** 1891-06

**Authors:** 


### INDIAN VETERINARY JOURMAL.

Our regret, at the termination of the *Quarterly Journal of Veterinary Science* in India, with the last number of vol. 8, October 1890, was sincere, as we have looked with interest on the fruitful endeavors, of the talented Veterinarians of the Army in India, in their deversified work with elephants, camels, horses and cattle, among a people, which has furnished apt students, but which was difficult to reach on account of the entire difference of language. The labor incumbent upon the English Veterinarian in India, has been evident in the *etymology* of their materia medica; to reach even the English speaking natives, they have been obliged to use native words, both for diseases and for drugs used as remedies. From the pen of John H. Steel, the editor of the Journal, we received some of the most valuable additions to veterinary literature that have been published in recent years.

A new Journal has appeared, the *Indian Veterinary Journal*. A new Quarterly Journal devoted to veterinary matters, published in Urdu, edited by Henry I. Pease, M. R. C. V. S., London.

Army Veterinary Department, Veterinary Surgeon to the Punjab Government, and Professor of Pathology, Lahore Veterinary School.

The first number has reached us in hieroglyphics, and from our point of view, only valuable as indicating the advance of the profession in that country, for the volume of 70 pages is printed in Hindostanee, it has the appearance of each page being a wood cut. The title, with a picture of a Centaur, is evidently on the last page, and the writing goes backwards.

